# The Embassy of Good Science – a community driven initiative to promote ethics and integrity in research

**DOI:** 10.12688/openreseurope.14422.1

**Published:** 2022-03-02

**Authors:** Marc van Hoof, Natalie Evans, Giulia Inguaggiato, Ana Marušić, Bert Gordijn, Kris Dierickx, David van Zeggeren, Harald Dunnik, Alexander Gesinn, Lex Bouter, Guy Widdershoven, EnTIRE and VIRT2UE consortia

**Affiliations:** 1 Department of Ethics, Law and Humanities, Amsterdam UMC, Vrije Universiteit, Amsterdam, Noord-Holland, 1081 HV, The Netherlands; 2 Department of Research in Biomedicine and Health, University of Split School of Medicine, Split, Split-Dalmatia, HR-21000, Croatia; 3 Institute of Ethics, Dublin City University, Dublin, Leinster, 9, Ireland; 4 Interfaculty Center for Biomedical Ethics and Law, KU Leuven, Leuven, 3000, Belgium; 5 Momkai BV, Amsterdam, Noord-Holland, 1013 NJ, The Netherlands; 6 Gesinn.it, Schwarzenfeld, 92521, Germany; 7 Department of Philosophy, Amsterdam UMC, Vrije Universiteit, Amsterdam, Noord-Holland, 1081 HV, The Netherlands

**Keywords:** Research integrity, research ethics, responsible conduct of research, open source, policy, education

## Abstract

The Embassy of Good Science ( https://www.embassy.science) aims to improve research integrity and research ethics by offering an online, open, 'go-to' platform, which brings together all information on research integrity and research ethics relevant for researchers, and makes that information accessible, understandable, and appealing. It effectively organizes and describes research integrity and research ethics guidelines, educational materials, cases, and scenarios. The Embassy is wiki-based, allowing users to add -- when logged in with their ORCID researcher id -- new information, and update and refine existing information. The platform also makes the research integrity and research ethics community visible and accessible in pages dedicated to relevant initiatives, news and events. Therefore, the Embassy enables researchers to find useful guidance, rules and tools to conduct research responsibly. The platform empowers researchers through increased knowledge and awareness, and through the support of the research integrity and research ethics community. In this article we will discuss the background of this new platform, the way in which it is organized, and how users can contribute.

## Plain language summary

The Embassy of Good Science ( https://www.embassy.science) aims to improve the conduct of research in Europe and beyond by presenting information of research integrity and ethics in an accessible, understandable and appealing way. The Embassy is wiki-based, allowing anyone to contribute. The platform also brings the research community together in novel ways in a dedicated community section. Therefore, The Embassy enables researchers to find useful guidance, rules and tools to conduct research responsibly. The platform empowers researchers through increased knowledge and awareness, and through the support of the research integrity and research ethics community. In this article we will discuss the background of this new platform, the way in which it is organized and how users can contribute.

## Background

The trustworthiness of research results is increasingly being questioned ^1^. In practice, research often leads to results that cannot be reproduced ^2– 5^. Several internal and external factors of human, operational and methodological origins have been identified ^6^. However, their interplay is anything but clear ^7, 8^. There is much attention to the classic triad of fabrication, falsification, and plagiarism, partly in response to high profile cases of misconduct ^9– 12^. However, other factors such as *'sloppy science'* and *'questionable research practices'* seem to have more effect and impact on research and researchers because of the sheer volume of these “small” misdemeanours ^13^. Researchers sometimes experience inadequate support from the institutions where they work in ensuring the quality of research. They are often promoted based on superficial outcome measures. The number of publications (‘ *publish or perish*’ ^14^), impact factors, citations, patents and the grants received dominate the evaluation and reward of researchers and science ^15^. The competition between researchers themselves is reinforced by the limited number of places that are available. Within this competitive system, the same researchers are also expected to act as independent judges of the work produced in their field; prior to publication or when awarding a grant, they assess the work of their direct competitors. It seems that individual poor research practices can be associated with common pitfalls and psychological coping mechanisms, adequately captured in the mnemonic TRAGEDIES ( *Temptation, Rationalization, Ambition, Group and authority pressure, Entitlement, Deception, Incrementalism, Embarrassment and Stupid systems*) ^16^.

Without denying current day problems, we are convinced that most researchers want to do their job well, but sometimes lack information about how to recognise the perverse incentives and pressures experienced in hyper-competitive academic environments, or how to find solutions to specific day-to-day practical, methodological and ethical questions. " *The Embassy of Good Science*" ( https://www.embassy.science) ^17^ is at the service of every researcher who wants to build up a practice of good research both by increasing knowledge and awareness of the best guidance and practices in research integrity and ethics and by sharing and collaboratively building new knowledge. We do this, for example, by making initiatives within the biomedical sciences easy to find and understand. In the case of REWARD ^18^, which aims to reduce waste in (medical) science and promote rigor and reproducibility, we sum up its best practices and make them shareable. The platform has been developed in consultation with stakeholders across Europe, whose needs and preferences are reflected in the platform’s content.

## Sharing knowledge, experiences and good practices

The purpose of the Embassy of Good Science is both to inform researchers and to enable them to share experiences and good practices surrounding research integrity and ethics. The Embassy brings together, and makes smart connections between, relevant guidelines and regulations, cases and scenarios, and teaching materials. ‘Theme’ pages provide engaging, bite-sized introductions to single topics related to good research practices, research principles, or misbehaviours. Complex guidance and practices are made accessible by describing why they are important, for whom they are important, and how can they guide the practice of research. Essentially, these theme pages provide the context in which all of the relevant resources on that topic across the platform are brought together and made usable and understandable. Users interested in, for instance ‘p-hacking’, ‘predatory publishing’, or ‘research virtues’, can read a Theme page of the topic, which links to resources (relevant guidance, online training, and misconduct cases) about the topic across The Embassy. Technically, specific information can be filtered and compared per discipline and country. In this way it is possible to organize a wide range of background information, examples of good practices, cases, (inter) national guidelines and regulations.

A dedicated ‘Training’ section provides access to free online training materials and courses, but also gives information about opportunities to follow more traditional in-person courses on integrity issues experienced in practice. The Embassy offers material that can be used by universities to provide training to researchers, (data) managers and other staff. Bespoke ‘Guides’ for training courses can also be constructed and made openly available directly on the platform. Guides consist of step-by-step instruction pages, describing individual exercises or activities, which users can make and group together themselves. The training guide for the European Commission funded VIRT2UE train-the-trainer programme, for example, is presented on The Embassy. VIRT2UE is a blended learning programme which gives trainees the knowledge and skills to conduct a research integrity course from a virtue ethics perspective. The VIRT2UE guide contains separate instruction pages for four series of e-learning modules and individual instruction pages detailing how to facilitate in person participatory exercises. In line with the ethos of the platform, VIRT2UE takes an aspirational approach, and focuses on what it means to be a good and virtuous researcher. The core of the training is to promote reflection on personal attitudes and practices.

The platform also makes the research integrity and ethics communities visible. Projects and consortia can inform, via The Embassy, an existing audience interested in research integrity and ethics about their initiatives, news and events. The repository function allows them to store their materials – such as reports, guidelines, leaflets - for the long term, allowing smaller initiatives to be represented online on The Embassy’s ‘Initiatives’ page rather than building their own websites from scratch. 

## Open science

The open-source platform is technically based on and inspired by Wikipedia (Wikimedia Foundation, USA). With approximately 47 million edits in November 2019 alone, and donations of 35 million dollars per year, this is a proven safe, scalable and well-maintained open-source system. Wikipedia is consulted 21 billion times a month worldwide ^19^. Where there has been discussion for years about the reliability of Wikipedia’s content, studies show that it is comparable to traditional encyclopaedias ^20, 21^. A review of specialist content, such as content from pharmacology ^22^, shows a similar picture. Meanwhile, with a total of approximately 50 million articles ^23^, it has most likely become the largest encyclopaedia in human history.

Just like with Wikipedia, the entire content of The Embassy is freely accessible to everyone. In addition, the content can be supplemented and adjusted by users. The only requirement for making an adjustment is an ORCID account (Open Researcher and Contributor ID, ORCID Inc.). Currently, more than 7 million researchers worldwide already have such an ORCID ^24^. To guarantee quality, all changes and additions can be traced at all times and, if necessary, corrected.

An important technical addition to the platform is the Semantic Mediawiki ^25^ extension. This makes it possible to organize content in a smart way (" *semantic web*"). The information is easily searchable and different sources of information can be linked to each other. The system works well for people and machines, according to the FAIR principles ^26^. This also makes it possible to give automated systems access to the knowledge that is collected on the platform. A complete overview of the open-source software used can be found on the platform itself ^27^.

On a wiki-platform it can be difficult for users to create and edit content. A core group of experts provide most of the content and changes to Wikipedia ^28^. To lower the threshold, increase usability and be inclusive, the user interface of The Embassy has been rebuilt from the ground up. It has been designed to be intuitive. We hope that ‘passive’ users (readers) are encouraged to submit and edit content (see the video). In the future, substantial digital contributions will be provided with a Digital Object Identifier (DOI) so that the content can be included in the scholarly literature and can be cited. Such micro publications ^29^ create the ability to transparently summarize, assess, discuss, verify, revoke, mix and expand information and knowledge.

The specific extensions and adjustments made by software developers within this project are also open source ^30^. This means that other organizations can reuse elements of the platform in a different configuration, for example by changing the design, structure and content.

## The online community moderates itself

Unlike other scientific platforms such as ResearchGate ^31^ (Berlin, Germany), The Embassy is not profit making. Compared to, e.g. Retraction Watch ^32^, users have the freedom to moderate the content and submit content themselves. The tone of The Embassy is also positive. We believe that " *Naming and shaming*" of individuals does not contribute to improving research practice or culture. The first content has been developed by the European Commission funded projects EnTIRE ^33^ and VIRT ^2^UE ^34^. In the coming years, the intention is that the online community will gradually take on further development and moderation. Just as with Wikipedia, there is a risk of incidents of online vandalism ^35^. This is expected to remain a limited problem. During the first year since going live, amongst a total of 7000 edits, just one edit could be considered as non-substantial ‘tinkering’. Clear versioning and tracked changes ensure good levels of control and enables editors to undo changes easily. In the case of Wikipedia, a study showed that about half of all entries that were vandalised and had a deletion of more than 90% were repaired within three minutes, while the overall average repair time was 8 days ^36^. A linked ORCID account ensures that the level and field of expertise of community members can be inspected, where relevant, when contributions are verified. Also, in cases of vandalism and a continued breach of the terms and conditions of the platform, users can be selectively blocked based on their ORCID.

## Conclusion

The Embassy of Good Science is a platform that has the potential to inform and engage the research community on research integrity and ethics. The platform aims to support individual researchers, but also, thorough increased awareness and action, to incrementally improve the trustworthiness of research and the culture in which research is conducted.

## Data availability

No data is associated with this article.

### Software availability

Platform available at: www.embassy.science

Front-end design elements: https://github.com/the-embassy-of-good-science

Archived front-end design elements: https://doi.org/10.5281/zenodo.5925902 ^37^

Back-end design elements: https://github.com/the-embassy-of-good-science/the-embassy-platform

Archived back-end design elements: https://doi.org/10.5281/zenodo.5925930 ^38^

License: MediaWiki and The Embassy are licensed under the terms of the GNU General Public License, version 2 or later. Derivative works and later versions of the code must be free software licensed under the same or a compatible license. This includes "extensions" that use MediaWiki functions or variables; see https://www.gnu.org/licenses/gpl-faq.html#GPLAndPlugins for details. For the full text of version 2 of the license, see: https://www.gnu.org/licenses/gpl-2.0.html. For full details of the licensing see: https://github.com/the-embassy-of-good-science/the-embassy-platform/blob/master/COPYING.
