# The Embassy of Good Science – a community driven initiative to promote ethics and integrity in research

**DOI:** 10.12688/openreseurope.14422.2

**Published:** 2023-01-12

**Authors:** Marc van Hoof, Natalie Evans, Giulia Inguaggiato, Ana Marušić, Bert Gordijn, Kris Dierickx, David van Zeggeren, Harald Dunnik, Alexander Gesinn, Lex Bouter, Guy Widdershoven, EnTIRE and VIRT2UE consortia

**Affiliations:** 1 Department of Ethics, Law and Humanities, Amsterdam UMC, Vrije Universiteit, Amsterdam, Noord-Holland, 1081 HV, The Netherlands; 2 Department of Research in Biomedicine and Health, University of Split School of Medicine, Split, Split-Dalmatia, HR-21000, Croatia; 3 Institute of Ethics, Dublin City University, Dublin, Leinster, 9, Ireland; 4 Interfaculty Center for Biomedical Ethics and Law, KU Leuven, Leuven, 3000, Belgium; 5 Momkai BV, Amsterdam, Noord-Holland, 1013 NJ, The Netherlands; 6 Gesinn.it, Schwarzenfeld, 92521, Germany; 7 Department of Philosophy, Amsterdam UMC, Vrije Universiteit, Amsterdam, Noord-Holland, 1081 HV, The Netherlands

**Keywords:** Research integrity, research ethics, responsible conduct of research, open source, policy, education

## Abstract

The Embassy of Good Science ( https://www.embassy.science) aims to improve research integrity and research ethics by offering an online, open, 'go-to' platform, which brings together information on research integrity and research ethics and makes that information accessible, understandable, and appealing. It effectively organizes and describes research integrity and research ethics guidelines, educational materials, cases, and scenarios. The Embassy is wiki-based, allowing users to add -- when logged in with their ORCID researcher id -- new information, and update and refine existing information. The platform also makes the research integrity and research ethics community visible and more accessible in pages dedicated to relevant initiatives, news and events. Therefore, the Embassy enables researchers to find useful guidance, rules and tools to conduct research responsibly. The platform empowers researchers through increased knowledge and awareness, and through the support of the research integrity and research ethics community. In this article we will discuss the background of this new platform, the way in which it is organized, and how users can contribute.

## Plain language summary

The Embassy of Good Science ( https://www.embassy.science) aims to improve the conduct of research in Europe and beyond by presenting information on research integrity and ethics in an accessible, understandable and appealing way. The Embassy is wiki-based, allowing anyone to contribute. The platform also brings the research community together in novel ways in a dedicated community section. The Embassy, therefore, enables researchers to find useful guidance, rules and tools to conduct research responsibly. The platform empowers researchers through increased knowledge and awareness, and through the support of the research integrity and ethics community. In this article we will discuss the background of this new platform, the way in which it is organized, and how users can contribute.

## Background

The trustworthiness of research results is increasingly being questioned ^1^. In practice, research results often cannot be reproduced ^2– 6^. Several internal and external factors of human, operational and methodological origins have been identified ^7, 8^. However, the importance of each is unclear. There is much attention to the classic triad of fabrication, falsification, and plagiarism, partly in response to high profile cases of misconduct ^9– 12^. However, *'sloppy science'* and *'questionable research practices'* might have more impact on research and researchers because of the sheer frequency of these “small” misdemeanours ^13– 15^. Whilst reproducibility is a clear concern in quantitative research, trustworthiness is also an issue for qualitative research, as it is not always clear how findings are supported by the data and how conclusions are drawn.

*The Embassy of Good Science* ( https://www.embassy.science) is an initiative that aims to improve the trustworthiness and integrity of research by supporting researchers to avoid questionable research practices and develop responsible research practices, both by increasing knowledge and awareness of the best guidance and practices in research integrity and ethics and by sharing and collaboratively building new knowledge ^16^. The Embassy is a wiki-based platform, allowing users to add - when logged in with their ORCID researcher id (Open Researcher and Contributor ID, ORCID Inc.) - new information, and update and refine existing information. The platform and its initial content have been developed, from 2017 to 2021, by the European Commission funded projects EnTIRE ^17^ and VIRT ^2^UE ^18^. The development process included an extensive consultation with stakeholders across Europe, whose needs and preferences related to research integrity and ethics are reflected in the platform’s design and content ^19^.

## Sharing knowledge, experiences and good practices

**Figure 1.**
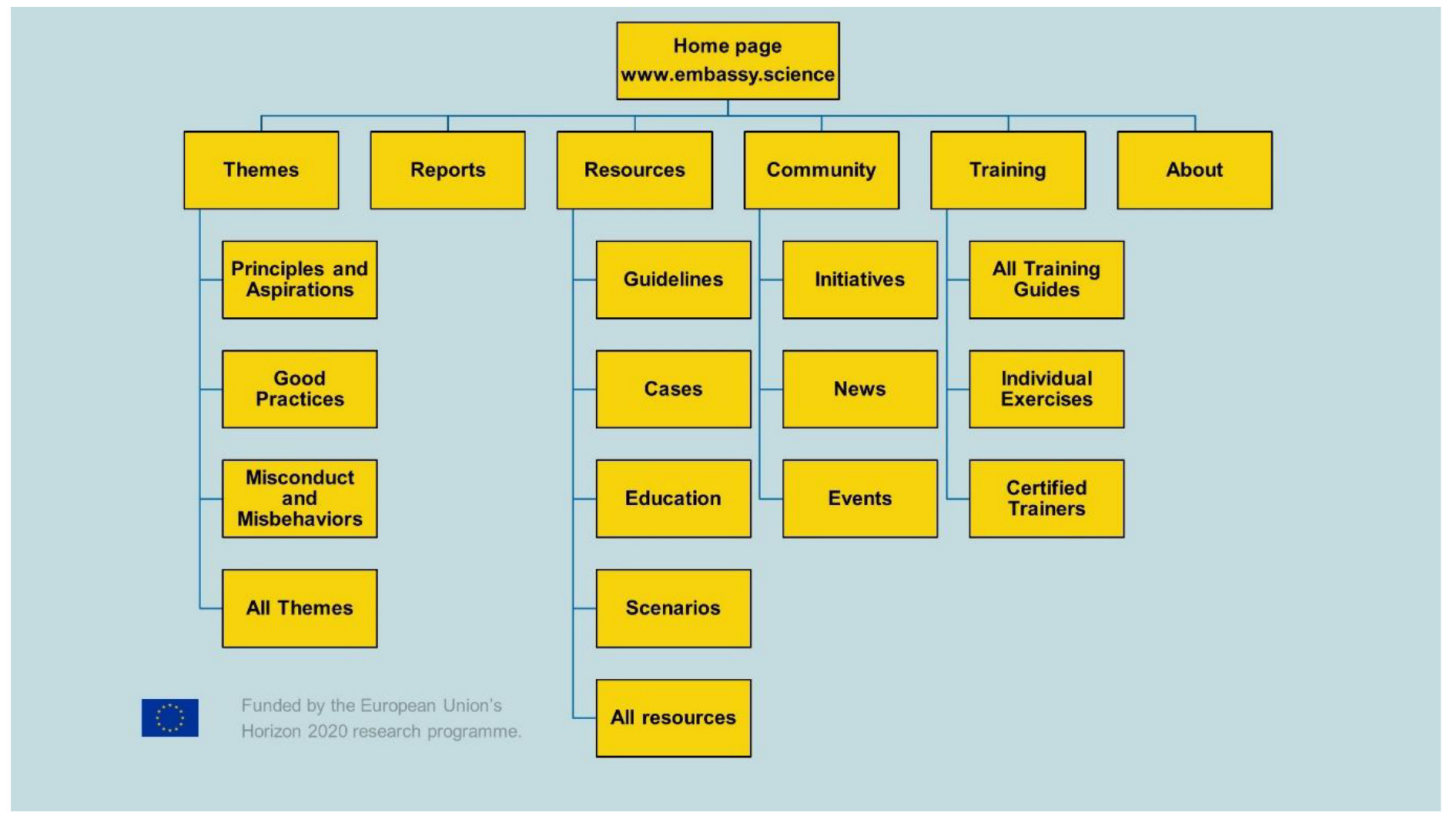
Site map of The Embassy’s permanent structure.

The Embassy brings together, and makes smart connections between, relevant research integrity and ethics guidelines and regulations, cases and scenarios, educational materials, and initiatives. Figure 1. provides a site map of the platform reflecting the permanent architecture of the site (within each of these categories, users are able to make new pages and hierarchies using the wiki functionalities). The content of the Embassy is organized into: ‘Themes’, ‘Reports’, ‘Resources’, ‘Community’, ‘Training’, and ‘About’.

‘Themes’ are pages which provide engaging, bite-sized introductions to single topics related to good research practices, research principles, or misbehaviours. Complex topics are made accessible by describing why they are important, for whom they are important, and what might be considered good practice examples. Essentially, each theme page provides the context in which all of the relevant resources on that topic across the Embassy platform are brought together and made usable and understandable. Users interested in, for instance ‘p-hacking’, ‘predatory publishing’, or ‘research virtues’, can read a Theme page on the topic, which links to resources (e.g., relevant guidance, online training, and misconduct cases) about the topic across The Embassy.

‘Reports’ describe the status of research infrastructure and governance across Europe. A semantic mediawiki tool which searches in and compares relevant topics across countries on the platform keeps these report cards up-to-date.

‘Resources’ represent relevant and openly available research integrity and ethics guidelines, cases, educational materials, and hypothetical scenarios. These resources were either systematically collected and described by the EnTIRE consortium ^20– 23^ or, in the case of the hypothetical scenarios, developed from scratch. Each resource is described and tagged in terms of what it is about and for whom it is important, enabling convenient filtering and search retrieval.

‘Community’ makes the research integrity and ethics communities visible. Projects and consortia can inform, via The Embassy, an existing user base interested in research integrity and ethics about their initiatives, news and events. The initiatives section allows individual projects to create a long-term repository of materials – such as reports, guidelines, leaflets, articles - allowing smaller initiatives to be represented online without the need to build their own websites from scratch.

‘Training’ provides access to free online training guides and information about opportunities to follow more traditional in-person courses. The Embassy offers material that can be used by universities to provide training to researchers, research integrity and ethics teachers, managers and other staff. Bespoke ‘Guides’ for training courses can also be constructed by users and made openly available on the platform. Guides consist of step-by-step instruction pages, describing how to do individual exercises or activities, which users can make and group together themselves. The training guide for the European Commission funded VIRT ^2^UE train-the-trainer programme, for example, is presented on The Embassy. VIRT ^2^UE is a blended learning programme which gives trainees the knowledge and skills to conduct a research integrity course from a virtue ethics perspective. The VIRT ^2^UE guide contains separate instruction pages for four series of e-learning modules and individual instruction pages detailing how to facilitate participatory exercises aimed at encouraging group reflection on research practice. In line with the ethos of the platform, VIRT ^2^UE takes a positive approach, and focuses on what it means to be a good and virtuous researcher.

Technically, content within each section can be tagged and searched. Theme pages and Resources can be filtered by, for example, discipline, target group, or country. In this way, it is possible to organize a wide range of information, examples of good practices, cases, and national and international guidelines and regulations.

The ‘About’ section provides information about the mission, management and funding of The Embassy platform, providing transparency about who is involved in the platform and how decisions are made regarding content. The non-profit ‘ Embassy of Good Science Foundation’ has been established to ensure the sustainability of the platform and further develop it based on the needs of users. Core funding is provided by involvement in European and national projects as a platform for the dissemination of results. The Foundation is supported by volunteer ‘ Ambassadors of Good Science’ who have substantially contributed to the structure, content and dissemination of the platform.

On every page, users can access The Embassy’s privacy statement, terms of service, take down policy and a contact form. The take down policy in particular provides details about the content that is, and is not, appropriate for The Embassy. The Embassy aims to provide a diversity of perspectives in relation to research integrity and ethics as long as they are not libellous or factually wrong.

An embedded evaluation button with questions specific for each section of The Embassy has also been integrated into the platform, allowing users to provide structured feedback (via answering a questionnaire) as well as report any platform bugs. As users need to actively opt to provide feedback via the embedded evaluation button, this has been little used to date. This embedded evaluation option does, however, provide the possibility to quickly respond to user feedback, e.g. two users indicated that they were unaware of the option to add or edit content. The wiki nature of the Embassy was subsequently stressed on The Embassy homepage.

The Embassy was initially developed to meet the needs of the European research community and its current content, and management, reflect this origin. Its reach, however, is global. Over time, user added content from outside of Europe will vastly increase the value of the platform by making diverse experiences and perspectives in relation to research integrity and ethics practices accessible for a global readership. The Foundation also hopes to attract more ‘Ambassadors’ from outside of Europe and have made some initial steps towards this by engaging with the African Research Integrity Network during the 7 ^th^ World Conference for Research Integrity held in Cape Town in 2022. The Embassy has been designed to ensure a good user experience across a range of devices, and mobile phones in particular, which facilitates access and contributions from low and middle income countries. Indeed, because anybody with an ORCID, access to the internet, time, and relevant research integrity and ethics knowledge, can develop content on the platform, the Embassy has the potential to breakdown traditional power imbalances in relation to knowledge transfer.

## Open science

The open-source platform is technically based on, and inspired by, Wikipedia (Wikimedia Foundation, USA). This is a proven safe, scalable and well-maintained open-source system. Wikipedia is consulted 21 billion times a month worldwide ^24^. Although there has been discussion for years about the reliability of Wikipedia’s content, studies show that it is comparable to traditional encyclopaedias ^25, 26^. A review of specialist content, such as content from pharmacology ^27^, shows a similar picture. Meanwhile, with a total of approximately 50 million articles ^28^, it has become the largest encyclopaedia in human history.

Just like with Wikipedia, the entire content of The Embassy is freely available and the content can be supplemented and adjusted by users. The only requirement for making an adjustment is an ORCID account. Currently, more than 7 million researchers worldwide already have such an ORCID ^29^. To guarantee quality, all changes and additions can be traced at all times and, if necessary, corrected. Furthermore, users are acknowledged by name at the end of pages to which they contribute, allowing for full transparency.

An important technical addition to the platform is the Semantic Mediawiki ^30^ extension. This makes it possible to organize content in a smart way (" *semantic web*"). The information is easily searchable and different sources of information can be linked to each other. The system works well for people and machines, according to the FAIR principles ^31^. This also makes it possible to give automated systems access to the knowledge that is collected on the platform. A complete overview of the open-source software used for The Embassy can be found on the platform itself ^32^.

On a wiki-platform it can be difficult for users to create and edit content. A core group of experts provide most of the content and changes to Wikipedia ^33^. To lower the threshold, increase usability, and be inclusive, the user interface of The Embassy has been rebuilt from the ground up. It has been designed to be intuitive. We hope that ‘passive’ users (readers) are encouraged to submit and edit content (see the video). In the future, substantial digital contributions will be provided with a Digital Object Identifier (DOI) so that the content can be included in the scholarly literature and can be cited. Such micro publications ^34^ create the ability to transparently summarize, assess, discuss, verify, revoke, mix and expand information and knowledge. If all small changes to content are publicised, it allows for full transparency. We will also be able to measure the types of additions (few and large, or small and numerous) which lead to the incremental development of research integrity and ethics knowledge.

The specific extensions and adjustments made by software developers within this project are also open source ^35^. This means that other organizations can reuse elements of the platform in a different configuration, for example by changing the design, structure and content.

## The online community moderates itself

Unlike other scientific platforms such as ResearchGate ^36^ (Berlin, Germany), The Embassy is not a commercial enterprise. Compared to other websites which aim to improve research integrity and ethics by sharing relevant information or cases (e.g. The Office for Research Integrity ( https://ori.hhs.gov/), The National Ethics Center ( https://nationalethicscenter.org/), the Online Ethics Center ( www.onlineethics.org), or Retraction Watch ( https://retractionwatch.com/)), The Embassy’s users have the freedom to moderate and add content themselves. The emphasis of the Embassy is positive, highlighting initiatives to improve research practices whilst also raising awareness about the research practices which might be considered ‘misconduct’ or ‘misbehavior’.

In the coming years, the intention is that the online community will gradually take on further development and moderation. This process will be guided by The Embassy of Good Science Foundation. A core team of Ambassadors are currently responsible for checking recent changes and rolling back any unsuitable changes and making minor content edits for style.

Just as with Wikipedia, there is of course a risk of incidents of online vandalism ^37^, however this is expected to remain a limited problem. During the first year after going live, amongst a total of 7000 edits, just one edit could be considered as non-substantial ‘tinkering’. Clear versioning and tracked changes ensure good levels of control and enable editors to undo changes easily. New additions are made by the community at least weekly, and recent changes can be followed here: Recent changes - The Embassy of Good Science. Changes are linked to contributors’ ORCID accounts, therefore community members can check contributors’ level of expertise. Also, in cases of vandalism or other substantial breaches of the terms and conditions of the platform, users can be selectively blocked based on their ORCID.

## Conclusion

The Embassy of Good Science is a platform that has the potential to inform and engage the research community on research integrity and ethics. The platform aims to support individual researchers, but also, thorough increased awareness and action, to incrementally improve the trustworthiness of research and the culture in which research is conducted.

## Data Availability

No data is associated with this article. Platform available at: www.embassy.science Front-end design elements: https://github.com/the-embassy-of-good-science Archived front-end design elements: https://doi.org/10.5281/zenodo.5925902 ^38^ Back-end design elements: https://github.com/the-embassy-of-good-science/the-embassy-platform Archived back-end design elements: https://doi.org/10.5281/zenodo.5925930 ^39^ License: MediaWiki and The Embassy are licensed under the terms of the GNU General Public License, version 2 or later. Derivative works and later versions of the code must be free software licensed under the same or a compatible license. This includes "extensions" that use MediaWiki functions or variables; see https://www.gnu.org/licenses/gpl-faq.html#GPLAndPlugins for details. For the full text of version 2 of the license, see: https://www.gnu.org/licenses/gpl-2.0.html. For full details of the licensing see: https://github.com/the-embassy-of-good-science/the-embassy-platform/blob/master/COPYING.
