# Supplementary material for: The Embassy of Good Science – a community driven initiative to promote ethics and integrity in research
Source: Open Res Eur. 2023 Jan 12;2:27. Originally published 2022 Mar 2. [Version 2] doi: 10.12688/openreseurope.14422.2 (PMC10521075; doi:10.12688/openreseurope.14422.2)
Supplement: Peer review history

# Reviewer report: Reviewer response for version 2

**DOI:** 10.21956/openreseurope.16659.r30621

**Published:** 2023-03-10

**Authors:** Jennifer Byrne

**Affiliations:** 1 Faculty of Medicine and Health, The University of Sydney, Sydney, Australia

Where applicable, are recommendations and next steps explained clearly for others to follow? (Please consider whether others in the research community would be able to implement guidelines or recommendations and/or constructively engage in the debate)

Partly

Does the article adequately reference differing views and opinions?

Partly

Are all factual statements correct, and are statements and arguments made adequately supported by citations?

Partly

Is the rationale for the Open Letter provided in sufficient detail? (Please consider whether existing challenges in the field are outlined clearly and whether the purpose of the letter is explained)

Yes

Is the Open Letter written in accessible language? (Please consider whether all subject-specific terms, concepts and abbreviations are explained)

Yes

Reviewer Expertise:

Research integrity, biobanking, cancer genetics

I confirm that I have read this submission and believe that I have an appropriate level of expertise to confirm that it is of an acceptable scientific standard.

# Reviewer report: Reviewer response for version 2

**DOI:** 10.21956/openreseurope.16659.r30619

**Published:** 2023-01-20

**Authors:** Izak van Zyl

**Affiliations:** 1 Cape Peninsula University of Technology, Cape Town, South Africa

I appreciate how the authors responded to my comments. I'm happy to accept this as a final version.

Where applicable, are recommendations and next steps explained clearly for others to follow? (Please consider whether others in the research community would be able to implement guidelines or recommendations and/or constructively engage in the debate)

Partly

Does the article adequately reference differing views and opinions?

No

Are all factual statements correct, and are statements and arguments made adequately supported by citations?

Partly

Is the rationale for the Open Letter provided in sufficient detail? (Please consider whether existing challenges in the field are outlined clearly and whether the purpose of the letter is explained)

Partly

Is the Open Letter written in accessible language? (Please consider whether all subject-specific terms, concepts and abbreviations are explained)

Yes

Reviewer Expertise:

Ethics; communication studies; transdisciplinary studies

I confirm that I have read this submission and believe that I have an appropriate level of expertise to confirm that it is of an acceptable scientific standard.

# Reviewer report: Reviewer response for version 2

**DOI:** 10.21956/openreseurope.16659.r30620

**Published:** 2023-01-13

**Authors:** Donald Sharpe

**Affiliations:** 1 University of Regina, Regina, Canada

No additional comments.

Where applicable, are recommendations and next steps explained clearly for others to follow? (Please consider whether others in the research community would be able to implement guidelines or recommendations and/or constructively engage in the debate)

Yes

Does the article adequately reference differing views and opinions?

Partly

Are all factual statements correct, and are statements and arguments made adequately supported by citations?

Yes

Is the rationale for the Open Letter provided in sufficient detail? (Please consider whether existing challenges in the field are outlined clearly and whether the purpose of the letter is explained)

Yes

Is the Open Letter written in accessible language? (Please consider whether all subject-specific terms, concepts and abbreviations are explained)

Yes

Reviewer Expertise:

Research methodology, statistics, ethics

I confirm that I have read this submission and believe that I have an appropriate level of expertise to confirm that it is of an acceptable scientific standard.

# Reviewer report: Reviewer response for version 1

**DOI:** 10.21956/openreseurope.15567.r29460

**Published:** 2022-06-21

**Authors:** Izak van Zyl

**Affiliations:** 1 Cape Peninsula University of Technology, Cape Town, South Africa

This open letter discusses a ‘community-driven initiative’ that houses information about ethics and research integrity in a collaborative virtual space.

 I am unsure how the Embassy initiative can bring together “ALL information on research integrity and research ethics relevant for researchers” (emphasis added). The Embassy is wiki-based, meaning that the scope and quality of such information are determined by the scientific community, and more specifically, anyone with an ORCID and access to a computer, with the time, knowledge and energy to do so. This already implies a potential epistemological disconnect, on one hand, between the compilers and readers of the information, as well as a potential power imbalance, on the other. Who would be more inclined to read and contribute? All 26 Embassy founders are to my knowledge based in Europe. The open letter itself acknowledges that the “platform has been developed in consultation with stakeholders across Europe, whose needs and preferences are reflected in the platform’s content”. If it were to be inclusive, how does this initiative, then, engage communities from the global South? How does it create meaningful opportunities to amplify historically marginalised (scientific) voices?

 I find the claim about irreproducibility to be misleading, at best. The concept of reproducibility is not germane in the qualitative, interpretivist, constructivist or critical paradigms. Claiming a universal reproducibility in “science” reinforces the epistemological disconnect (or even bias) I raise above. Then, the article tends to rely on older sources to substantiate some serious ‘current’ claims – like that of trustworthiness and reproducibility, for example.

 I do not know what is meant by the claim that “their interplay is anything but clear”. Here, the authors write that human, operational and methodological factors affect the veracity of the research endeavour, which is obviously true. But that their interplay is unclear is not substantiated. Good science  – anywhere – is reflective, critical science. This does not mean that instances of ‘bad science’ will not occur. It means that interplaying factors do not go unnoticed, and many scholars devote meta-analyses toward these.

 I do not find the claim that researchers are “often promoted based on superficial outcome measures” to be substantive, and the claim that researchers are rewarded and evaluated purely on measurable outcomes is based on a single study from Hong Kong. Of course, it happens in many universities across the world, but to claim ‘universal superficiality’ is simply false. The link to the so-called TRAGEDIES model is both legitimate and interesting. However, even this model is up for scrutiny as a ‘comment’ and not a peer-reviewed resource.

 The Embassy’s vision to share knowledge, experiences and good practices is laudable but ambitious. I am not familiar with this initiative, but I can anticipate it will need critical mass to stimulate uptake. It builds on the Wikipedia model, but this model presents two challenges: 1) how is it economically viable as free and open-source software (FOSS); and 2) how does it guarantee factually correct content across multiple disciplines? In the case of the former, the Wikipedia model is not self-sustainable: it relies on donations. In the case of the latter, the authors use the term “corrected” to guarantee quality, but how will this be/is this done? I am not so concerned about online vandalism as I am about credibility, factuality and veracity. There are also many different interpretations of and approaches to ethics in research. I advocate for something like intersubjective ethics, but this is traditionally at odds with the biomedical model. How are these types of complexities accounted for?

 Overall, this is a noteworthy initiative and I look forward to engaging with it in future. It does, however, present serious complexities which I hope the authors can address in future open letters or publications. All the best with this initiative.

Where applicable, are recommendations and next steps explained clearly for others to follow? (Please consider whether others in the research community would be able to implement guidelines or recommendations and/or constructively engage in the debate)

Partly

Does the article adequately reference differing views and opinions?

No

Are all factual statements correct, and are statements and arguments made adequately supported by citations?

Partly

Is the rationale for the Open Letter provided in sufficient detail? (Please consider whether existing challenges in the field are outlined clearly and whether the purpose of the letter is explained)

Partly

Is the Open Letter written in accessible language? (Please consider whether all subject-specific terms, concepts and abbreviations are explained)

Yes

Reviewer Expertise:

Ethics; communication studies; transdisciplinary studies

I confirm that I have read this submission and believe that I have an appropriate level of expertise to confirm that it is of an acceptable scientific standard, however I have significant reservations, as outlined above.

# Response

**Published:** 2022-12-28

**Authors:** Natalie Evans

**Affiliations:** Amsterdam UMC, The Netherlands

We thank the reviewer for their nuanced and important comments which we have tried to answer below one by one:

1. Thank you for this important suggestion. As mentioned in the response to reviewer 2, we have removed the word ‘all’ from the sentence. The text now reads: 
  “which brings together information on research integrity and research ethics relevant for researchers, and makes that information accessible, understandable, and appealing.” 
2. Thank you also for this very important comment related to whose knowledge is prioritised. We have included the following text to emphasize the European origin of the platform content and the potential for inclusive knowledge production. 
  “The Embassy was initially developed to meet the needs of the European research community and its current content, and management, reflect this origin. Its reach, however, is global. Over time, user added content from outside of Europe will vastly increase the value of the platform by making diverse experiences and perspectives in relation to research integrity and ethics practices accessible for a global readership. The Foundation also hopes to attract more ‘Ambassadors’ from outside of Europe and have made some initial steps towards this by engaging with the African Research Integrity Network during the 7th World Conference for Research Integrity held in Cape Town in 2022. The Embassy has been designed to ensure a good user experience across a range of devices, and mobile phones in particular, which facilitates access and contributions from low and middle income countries.  
  Indeed, because anybody with an ORCID, access to the internet, time, and relevant research integrity and ethics knowledge can develop content on the platform, the Embassy has the potential to breakdown traditional power imbalances in relation to knowledge transfer.”
3. Thank you for this comment. We have kept in the statement about reproducibility because the so called ‘reproducibility crisis’ provides impetus for many research integrity initiatives. We have updated the references. We also include a sentence about trustworthiness of qualitative research: 
  “Whilst reproducibility is a clear concern in quantitative research, trustworthiness is also an issue for qualitative research, as it is not always clear how findings are supported by the data and how conclusions are drawn.”
4. Thank you for this comment, we have change the sentence to “However, the importance of each is unclear”.
5. Thank you for this comment, we have now removed this text from the paper as it is not immediately relevant for the introduction of The Embassy website.
6. Thank you for this comment. In response to point 1), we have provided some more information about the platform management and financial sustainability here: 
  “The ‘About’ section provides information about the mission, management and funding of The Embassy platform, ensuring transparency on who is involved in the platform and how decisions are made regarding content. The non-profit ‘Embassy of Good Science Foundation’ has been established to ensure sustainability of the platform and further develop it based on the needs of users. Core funding is provided by involvement in European and national projects as a platform for the dissemination of results. The Foundation is supported by volunteer  ‘Ambassadors of Good Science’ who have substantially contributed to the structure, content and dissemination of the platform.”  
  In response to point 2), we have provided additional information here: 
  “In the coming years, the intention is that the online community will gradually take on further development and moderation. This process will be guided by The Embassy of Good Science Foundation. A core team of Ambassadors are currently responsible for checking recent changes and rolling back any unsuitable changes and making minor content edits for style. Just as with Wikipedia, there is of course a risk of incidents of online vandalism, however this is expected to remain a limited problem. During the first year after going live, amongst a total of 7000 edits, just one edit could be considered as non-substantial ‘tinkering’. Clear versioning and tracked changes ensure good levels of control and enables editors to undo changes easily. New additions are made by the community at least weekly, and recent changes can be followed here: Recent changes - The Embassy of Good Science. Changes are linked to contributors’ ORCID accounts, therefore community members can check contributors’ level of expertise. Also, in cases of vandalism and substantial breaches of the terms and conditions of the platform, users can be selectively blocked based on their ORCID.” 
7. We also appreciate the reviewer’s hope for representation of diverse perspectives. More information about this is provided here: 
  “On every page, users can access The Embassy’s privacy statement, terms of service, take down policy and a contact form. The take down policy in particular provides details about the content that is, and is not, appropriate for The Embassy. The Embassy aims to provide a diversity of perspectives in relation to research integrity and ethics as long as they are not libellous or factually wrong.” 

# Reviewer report: Reviewer response for version 1

**DOI:** 10.21956/openreseurope.15567.r29463

**Published:** 2022-06-20

**Authors:** Donald Sharpe

**Affiliations:** 1 University of Regina, Regina, Canada

The paper does a good job of bringing to the attention of the research community the Embassy of Good Science, explaining its objectives and purpose. All of my comments are fairly minor, mostly observations as I read the paper.

 The abstract speaks to bringing together “all information on research integrity and research ethics relevant for researchers”. I am not sure “all information” can possibly be collected, especially given what we know changes constantly. The other issue with ethics is frequently there are no right or wrong answers but rather varying opinions. For example, is deception in research good or bad?

 The Background section made a few claims, all supported by citations, that nonetheless I struggled with. “Research often leads to results that cannot be reproduced”. What does “often” mean here? Although some efforts to replicate classic findings have failed, many have not. How one defines success or failure here is complicated. And no one has attempted to sample all research to see what the failure rate is.

 “[Researchers] are often promoted based on superficial outcome measures… number of publications… impact factors, citations, patents, and the grants received”. I am not sure that many researchers (including myself) would regard these outcome measures to be superficial. Getting publications is hard. Impact factors are a measure of research impact to some extent. Citations do indicate one’s work is being read and incorporated into the literature. Success at obtaining grants and patents should be recognized.

 “Perverse incentives and pressures experienced in hyper-competitive academic environments”. That’s a bit strong. Academia has its challenges but many individuals enjoy being in academia, thrive on competition, and appreciate its benefits (monetary and otherwise).

 Open Science --- Wikipedia. Perhaps a bit more was said about Wikipedia than is needed here, but the comparison does explain what the authors are striving to achieve.

 Overall, interesting paper as an introduction to the Embassy of Good Science.

Where applicable, are recommendations and next steps explained clearly for others to follow? (Please consider whether others in the research community would be able to implement guidelines or recommendations and/or constructively engage in the debate)

Yes

Does the article adequately reference differing views and opinions?

Partly

Are all factual statements correct, and are statements and arguments made adequately supported by citations?

Yes

Is the rationale for the Open Letter provided in sufficient detail? (Please consider whether existing challenges in the field are outlined clearly and whether the purpose of the letter is explained)

Yes

Is the Open Letter written in accessible language? (Please consider whether all subject-specific terms, concepts and abbreviations are explained)

Yes

Reviewer Expertise:

Research methodology, statistics, ethics

I confirm that I have read this submission and believe that I have an appropriate level of expertise to confirm that it is of an acceptable scientific standard.

# Response

**Published:** 2022-12-28

**Authors:** Natalie Evans

**Affiliations:** Amsterdam UMC, The Netherlands

We thank the reviewer for their time and very useful observations. We respond to the comments one by one below:

1.  We have removed the word ‘all’ from the sentence. The text now reads “which brings together information on research integrity and research ethics relevant for researchers, and makes that information accessible, understandable, and appealing.”
2. Thank you for this important nuance. We have updated the references to include reproducibility studies in various disciplines. These indicate that more than half of results from psychology, social science, and preclinical cancer studies failed to replicate, which reflects our claim that research results often cannot be reproduced.
3. Thank you for this comment, we have now removed this text from the paper as it is not immediately relevant for the introduction of The Embassy website.
4. Thank you for this comment, we have now removed this text from the paper as it is not immediately relevant for the introduction of The Embassy website. 

# Reviewer report: Reviewer response for version 1

**DOI:** 10.21956/openreseurope.15567.r28684

**Published:** 2022-03-21

**Authors:** Jennifer Byrne

**Affiliations:** 1 Faculty of Medicine and Health, The University of Sydney, Sydney, Australia

The open letter by Van Hoof and colleagues provides a useful overview of the Embassy of Good Science and its structure and ambitions. The Embassy of Good Science promotes the principles and practices of good science through the provision of information, education and practical tools and deserves to be widely known. The letter is clearly written and easily understood. My comments are intended to clarify a small number of issues and suggest additions that would add to the letter’s impact.

 Major Comments:

1. The letter would greatly benefit from a summary diagram that mirrors the Embassy’s website structure. I found myself looking for this diagram as soon as I reached the “Sharing knowledge, experiences and good practices” section (page 3). Descriptions of theme pages, the training section, and statements such as “four series of e-learning modules” (page 3) would all be supported by a summary diagram.
2. “Compared to eg. Retraction Watch, users have the freedom to moderate the content and submit content themselves” (page 4). I was somewhat confused by the comparison with Retraction Watch, as Retraction Watch does not aim to serve the same purpose as the Embassy of Good Science. In this sense, it is perhaps not surprising that Retraction Watch has a different user engagement model.
3. “The tone of the Embassy is also positive” (page 4). When I read this statement, I expected that the Embassy of Good Science does not provide information about research misconduct, but when I visited the website, I found that this information was available. It may be helpful to specify that Embassy of Good Science does provide information about “Misconduct and misbehaviors” as one of 4 themes. Please see comment 1 about the need for a summary diagram.
4. Similarly “We believe that “Naming…” (page 4). Is there any published evidence to support the stated belief that “Naming and shaming of individuals does not contribute to improving research culture of practice”? The term “naming and shaming” is in my view overly simplistic and risks detracting from discussions of misconduct where perpetrators need to be identified.
5. The letter could give more information about (i) the user feedback received to date, (ii) if or how this has supported the development of the material offered by the Embassy of Good Science, and (iii) how often content will be reviewed and updated.
6. Similarly, the letter could describe research that is being done based on the Embassy of Good Science platform and its use and/or opportunities for future research (see for example minor comment 3 below).

Minor comments

1. No references were cited for the two consecutive statements “Researchers sometimes receive inadequate support…” and “They are often promoted based on….” (page 3).
2. “…The Embassy is freely available to everyone” (page 4): “to everyone” could be removed- while well intentioned, there are inevitable restrictions on access, notably the need for access to the internet and to material in English. Some reference could be made about accessibility of content via mobile devices or phones, as this might facilitate access by LMIC users.
3. Is there evidence that “micro publications create the ability to transparently summarise….”? (page 4). Could future research on the use of micro publications by the Embassy of Good Science add to the evidence base here?
4. “The first content has been developed by the European Commission funded” (page 4): It would be helpful to specify over which years EC funding was available. Please also note “VIRT ^2^UE’ as opposed to “VIRT2UE” elsewhere in the text.
5. Similarly, “in the coming years” (page 4): It would be helpful to specify over which timeframe the online community will “gradually take on further development and moderation”, and how this process is envisaged to work. Presumably, there will always be a role for some type of central oversight, so it would be helpful to provide more information about how any transition will occur, and to what extent the model will rely upon ongoing external funding.

Where applicable, are recommendations and next steps explained clearly for others to follow? (Please consider whether others in the research community would be able to implement guidelines or recommendations and/or constructively engage in the debate)

Partly

Does the article adequately reference differing views and opinions?

Partly

Are all factual statements correct, and are statements and arguments made adequately supported by citations?

Partly

Is the rationale for the Open Letter provided in sufficient detail? (Please consider whether existing challenges in the field are outlined clearly and whether the purpose of the letter is explained)

Yes

Is the Open Letter written in accessible language? (Please consider whether all subject-specific terms, concepts and abbreviations are explained)

Yes

Reviewer Expertise:

Research integrity, biobanking, cancer genetics

I confirm that I have read this submission and believe that I have an appropriate level of expertise to confirm that it is of an acceptable scientific standard, however I have significant reservations, as outlined above.

# Response

**Published:** 2023-01-10

**Authors:** Natalie Evans

**Affiliations:** Amsterdam UMC, The Netherlands

We thank the reviewer for their kind and useful comments and the time and expertise they have provided in this review. Below you will find our responses. *Major comments*

1. Thank you for this suggestion. Because The Embassy platform is a wiki and new pages and structures can be generated by users at any point, we did not initially offer a site map. However, we have now created a site map providing only the more permanent architecture of the site (See figure 1. in version 2).  We have included and referred to this figure in the section “Sharing knowledge, experiences and good practices”: To help the reader in their comprehension of the different sections of The Embassy platform, we have restructured this section to reflect the categories of the site map. We have also provided further information about the ‘Reports’ section of the platform, which was not yet online when the commentary was submitted. 
2. Thank you for the comment. The point we are trying to highlight here is the ability of users to add their own content, in contrast to other sites which aim to improve research integrity and ethics. To emphasize this, we have changed the wording to *: “ Unlike other scientific platforms such as ResearchGate (Berlin, Germany), The Embassy is not a commercial enterprise. Compared to other websites which aim to improve research integrity and ethics by sharing relevant information or cases (e.g. The Office for Research Integrity (https://ori.hhs.gov/), The National Ethics Center (https://nationalethicscenter.org/), the Online Ethics Center (www.onlineethics.org), or Retraction Watch (https://retractionwatch.com/)), The Embassy’s users have the freedom to moderate and add content themselves”. *
3. Thank you for the comment. We have changed the wording to: “The emphasis of the Embassy is positive, highlighting initiatives to improve research practices whilst also raising awareness about the research practices which might be considered ‘misconduct’ or ‘misbehavior’”.  
4. Thank you for this comment. Also in line with the comment above, we have changed the text from *: “The tone of The Embassy is also positive. We believe that " Naming and shaming" of individuals does not contribute to improving research practice or culture. The first content has been developed by the European Commission funded projects EnTIRE and VIRT 2UE” *to *“The emphasis of the Embassy is positive, highlighting initiatives to improve research practices whilst also raising awareness about the research practices which might be considered ‘misbehavior’ or ‘misconduct’.” *
5. Thank you for these comments. In answer to points 1 and 2, The Embassy was developed in consultation with stakeholders in which their research integrity and ethics support needs were identified and information about their preference for the online platform were explored. These consultations provided insights which directly influenced the design, navigation, content, and tone of the Embassy. This origin is now more thoroughly reflected in the Background section *: “The development process included an extensive consultation with stakeholders across Europe, whose needs and preferences related to research integrity and ethics are reflected in the platform’s design and content.” *Furthermore, an embedded evaluation button with questions specific for each section of The Embassy has been integrated into the platform, allowing users to provide structure feedback (via answering a questionnaire) as well as report any platform bugs. Information about this feedback mechanism (which has only had eight submissions to date) is now provided in the ‘Sharing knowledge, experiences and good practices’ section *: “An embedded evaluation button with questions specific for each section of The Embassy has also been integrated into the platform, allowing users to provide structured feedback (via answering a questionnaire) as well as report any platform bugs. As users need to actively opt to provide feedback via the embedded evaluation button, this has been little used to date. This embedded evaluation option does, however, provide the possibility to quickly respond to user feedback, e.g. two users indicated that they were unaware of the option to add or edit content. The wiki nature of the Embassy was subsequently stressed on The Embassy homepage.” *In answer to point 3, content is continually added by the community of users and new content is moderated daily. We realise that this is more important to emphasize than describing the number of edits on Wikipedia. To reflect this the text has been changed from: *“During the first year since going live, amongst a total of 7000 edits, just one edit could be considered as non-substantial ‘tinkering’. Clear versioning and tracked changes ensure good levels of control and enables editors to undo changes easily. In the case of Wikipedia, a study showed that about half of all entries that were vandalised and had a deletion of more than 90% were repaired within three minutes, while the overall average repair time was 8 days 36 . A linked ORCID account ensures that the level and field of expertise of community members can be inspected, where relevant, when contributions are verified. Also, in cases of vandalism and a continued breach of the terms and conditions of the platform, users can be selectively blocked based on their ORCID” *to *“In the coming years, the intention is that the online community will gradually take on further development and moderation. This process will be guided by The Embassy of Good Science Foundation. A core team of Ambassadors are currently responsible for checking recent changes and rolling back any unsuitable changes and making minor content edits for style. Just as with Wikipedia, there is of course a risk of incidents of online vandalism, however this is expected to remain a limited problem. During the first year after going live, amongst a total of 7000 edits, just one edit could be considered as non-substantial ‘tinkering’. Clear versioning and tracked changes ensure good levels of control and enables editors to undo changes easily. New additions are made by the community at least weekly, and recent changes can be followed here: Recent changes - The Embassy of Good Science. Changes are linked to contributors’ ORCID accounts, therefore community members can check contributors’ level of expertise. Also, in cases of vandalism or other substantial breaches of the terms and conditions of the platform, users can be selectively blocked based on their ORCID.” *
6. Currently, the relationship between research and The Embassy is that research is disseminated and made more understandable and practically useful through the Embassy. For example, the primary data collected for systematic reviews of policy documents, which once would have been difficult to access, are immediately available and fully described on the platform. The content from the following studies is available, described and made more accessible on The Embassy: ♦ Desmond, H., & Dierickx, K. (2021). Research integrity codes of conduct in Europe: Understanding the divergences. Bioethics, 35(5), 414-428; ♦ Hastings, RC., Labib, K. Lechner, IM. et al.; Rosie Hastings, Krishma Labib, Iris Lechner, Lex Bouter, Guy Widdershoven, Natalie Evans, on behalf of the EnTIRE Consortium, Guidance on research integrity provided by pan-European discipline-specific learned societies: A scoping review, *Science and Public Policy*, 2022;, scac067, https://doi.org/10.1093/scipol/scac067; ♦  Armond AC, Gordijn B, Lewis J, Hosseini M, Bodnár JK, Holm S, Kakuk P. A scoping review of the literature featuring research ethics and research integrity cases. BMC medical ethics. 2021;22(1):1-4; ♦ Pizzolato D, Abdi S, Dierickx K. Collecting and characterizing existing and freely accessible research integrity educational resources. Accountability in Research. 2020; 18;27(4):195-211.  This is represented in the additional information now given about ‘resources’ *: “‘Resources’ represent relevant and openly available research integrity and ethics guidelines, cases, educational materials, and hypothetical scenarios. These resources were either systematically collected and described by the EnTIRE consortium 20, 21, 22, 23 or, in the case of the hypothetical scenarios, developed from scratch. Each resource is described and tagged in terms of what it is about and for whom it is important, enabling convenient filtering and search retrieval.” *In the future, we hope that The Embassy will also be a source to be searched for policy documents, cases and educational materials related to research ethics and research integrity, a possibility which is facilitated by the platform’s advanced search and filtering functions.  

* Minor comments*

1. Thank you for pointing this out. We have now removed these statements. The rationale for the development of The Embassy is that sloppy science and questionable research practices are frequent and detrimental to science – this is enough. We do not need to go into the systemic and behavioural reasons for these practices.
2. Thank you for this important comment. Indeed, we put great efforts into ensuring a good user experience across a range of devices. We have changed the wording to *: “Just like with Wikipedia, the entire content of The Embassy is freely available and the content can be supplemented and adjusted by users” *and *“The Embassy was initially developed to meet the needs of the European research community and its current content, and management, reflect this origin. Its reach, however, is global. Over time, user added content from outside of Europe will vastly increase the value of the platform by making diverse experiences and perspectives in relation to research integrity and ethics practices accessible for a global readership. The Foundation also hopes to attract more ‘Ambassadors’ from outside of Europe and have made some initial steps towards this by engaging with the African Research Integrity Network during the 7th World Conference for Research Integrity held in Cape Town in 2022. The Embassy has been designed to ensure a good user experience across a range of devices, and mobile phones in particular, which facilitates access and contributions from low and middle income countries.  Indeed, because anybody with an ORCID, access to the internet, time, and relevant research integrity and ethics knowledge can develop content on the platform, the Embassy has the potential to breakdown traditional power imbalances in relation to knowledge transfer.“ *
3. Thank you for this comment, this is an important point. We have added to the text on micro publications and their contribution to transparency and measurability as follows: *“If all small changes to content are publically available, it allows for full transparency. We will also be able to measure the types of additions (few and large or small and numerous) which lead to the incremental development of research integrity and ethics knowledge.”  *
4. Thank you for asking for this clarification. We have changed the wording to *: “The platform and its initial content have been developed, from 2017 to 2021, by the European Commission funded projects EnTIRE 17 and VIRT2UE 18 . The development process included an extensive consultation with stakeholders across Europe, whose needs and preferences are reflected in the platform’s design and content.” *
5. Thank you for pointing this out. We have edited the document to ensure the 2 in VIRT2UE is now always superscript.
6. Thank you for this comment. We have provided the following additional details in the text *: “The ‘About’ section provides information about the mission, management and funding of The Embassy platform, ensuring transparency on who is involved in the platform and how decisions are made regarding content. The non-profit ‘ Embassy of Good Science Foundation’ has been established to ensure sustainability of the platform and further develop it based on the needs of users. Core funding is provided by involvement in European and national projects as a platform for the dissemination of results. The Foundation is supported by volunteer  ‘ Ambassadors of Good Science’ who have substantially contributed to the structure, content and dissemination of the platform. On every page, users can access The Embassy’s privacy statement, terms of service, take down policy and a contact form. The take down policy in particular provides details about the content that is, and is not, appropriate for The Embassy. The Embassy aims to provide a diversity of perspectives in relation to research integrity and ethics as long as they are not libellous or factually wrong. An embedded evaluation button with questions specific for each section of The Embassy has also been integrated into the platform, allowing users to provide structure feedback (via answering a questionnaire) as well as report any platform bugs. As users need to actively opt to provide feedback via the embedded evaluation button, this has been little used to date. This embedded evaluation option does, however, provide the possibility to quickly respond to user feedback, e.g. two users indicated that they were unaware of the option to add or edit content. The wiki nature of the Embassy was subsequently stressed on The Embassy homepage. The Embassy was initially developed to meet the needs of the European research community and its current content, and management, reflect this origin. Its reach, however, is global. Over time, user added content from outside of Europe will vastly increase the value of the platform by making diverse experiences and perspectives in relation to research integrity and ethics practices accessible for a global readership. The Foundation also hopes to attract more ‘Ambassadors’ from outside of Europe and have made some initial steps towards this by engaging with the African Research Integrity Network during the 7th World Conference for Research Integrity held in Cape Town in 2022. The Embassy has been designed to ensure a good user experience across a range of devices, and mobile phones in particular, which facilitates access and contributions from low and middle income countries.  Indeed, because anybody with an ORCID, access to the internet, time, and relevant research integrity and ethics knowledge, can develop content on the platform, the Embassy has the potential to breakdown traditional power imbalances in relation to knowledge transfer.”*
